# Overexpression of GPR39 contributes to malignant development of human esophageal squamous cell carcinoma

**DOI:** 10.1186/1471-2407-11-86

**Published:** 2011-02-25

**Authors:** Fajun Xie, Haibo Liu, Ying-Hui Zhu, Yan-Ru Qin, Yongdong Dai, Tingting Zeng, Leilei Chen, Changjun Nie, Hong Tang, Yan Li, Li Fu, Xin-Yuan Guan

**Affiliations:** 1 State Key Laboratory of Oncology in Southern China, Cancer Center, Sun Yat-Sen University, Guangzhou, PR China; 2 Department of Clinical Oncology, University of Hong Kong, Hong Kong, PR China; 3 Department of Clinical Oncology, the First Affiliated Hospital, Zhengzhou University, Zhengzhou, PR China; 4 Department of Clinical Oncology, the Cancer Hospital, Zhengzhou University, Zhengzhou, PR China

## Abstract

**Background:**

By using cDNA microarray analysis, we identified a G protein-coupled receptor, *GPR39*, that is significantly up-regulated in ESCC. The aim of this study is to investigate the role of GPR39 in human esophageal cancer development, and to examine the prevalence and clinical significance of GPR39 overexpression in ESCC.

**Methods:**

The mRNA expression level of *GPR39 *was analyzed in 9 ESCC cell lines and 50 primary ESCC tumors using semi-quantitative RT-PCR. Immunohistochemistry was used to assess GPR39 protein expression in tissue arrays containing 300 primary ESCC cases. In vitro and in vivo studies were done to elucidate the tumorigenic role of GPR39 in ESCC cells.

**Results:**

We found that GPR39 was frequently overexpressed in primary ESCCs in both mRNA level (27/50, 54%) and protein level (121/207, 58.5%), which was significantly associated with the lymph node metastasis and advanced TNM stage (*P *< 0.01). Functional studies showed that GPR39 has a strong tumorigenic ability. Introduction of *GPR39 *gene into ESCC cell line KYSE30 could promote cell proliferation, increase foci formation, colony formation in soft agar, and tumor formation in nude mice. The mechanism by which amplified GPR39 induces tumorigenesis was associated with its role in promoting G1/S transition via up-regulation of cyclin D1 and CDK6. Further study found GPR39 could enhance cell motility and invasiveness by inducing EMT and remodeling cytoskeleton. Moreover, depletion of endogenous GPR39 by siRNA could effectively decrease the oncogenicity of ESCC cells.

**Conclusions:**

The present study suggests that GPR39 plays an important tumorigenic role in the development and progression of ESCC.

## Background

Esophageal squamous cell carcinoma (ESCC), the major histological form of esophageal cancer, is one of the most aggressive malignancies with poor prognosis in the world, especially in the Northern part of China [1]. Like other types of solid tumors, the development of ESCC is also the accumulation of the abnormal expression of oncogenes and tumor suppressor genes (TSGs). Several genetic alterations have been associated with the development of ESCC including mutations of p53 and p16, amplification of cyclin D, c-myc, and EGFR, as well as allelic loss on chromosomes 3p, 5q, 8p, 9p, 9q, 13q, 17p, 18q, and 21q [2-5]. Our previous studies have characterized the common deletion regions at 3p and candidate TSGs within frequently deleted regions including *PLCD1 *and *PCAF *[6,7]. However, many genes associated with the development and progression of ESCC have not been characterized. To better understand the molecular mechanisms that underlie the ESCC development and progression, cDNA microarray was used to compare the gene expression profiles between 10 primary ESCC tumors and their paired non-tumorous tissues.

Among the 185 up-regulated genes, one gene named *GPR39 *drew our attention. GPR39 belongs to the G protein-coupled receptors (GPCRs) superfamily, which is the largest family of cell-surface molecules involved in signal transmission. It has been reported that GPR39 plays an important role in the regulation of gastrointestinal and metabolic function [8]. GPR39 receptor is now thought to be activated by Zn^2+ ^signals and may have other, as yet unidentified, cognitive ligands [9]. Moreover, GPR39 receptor also displays a strong ligand-independent signaling activity through Gα_12/13 _as well as Gα_q _[10,11]. A recent study suggests that overexpression of GPR39 may inhibit cell death induced by oxidative stress, endoplasmic reticulum (ER) stress, and activation of the caspase by Bax overexpression [12]. Emerging evidence indicates that G protein-coupled receptors are crucial players in cancer progression and metastasis [13,14], however, the role of GPR39 in cancer development remains unclear. In this study, we studied GPR39 expression pattern in ESCC. The tumorigenic function of GPR39 was demonstrated by both *in vitro *and *in vivo *assays. The tumorigenic mechanism of GPR39 was also addressed. In addition, the clinical significance of GPR39 overexpression in ESCC was investigated.

## Methods

### ESCC cell lines and specimens

Chinese ESCC cell line HKESC1 was kindly provided by Professor Srivastava (Department of Pathology, The University of Hong Kong, Hong Kong, China), and two Chinese ESCC cell lines (EC18 and EC109) were kindly provided by Professor Tsao (Department of Anatomy, The University of Hong Kong). Six Japanese ESCC cell lines (KYSE30, KYSE140, KYSE180, KYSE410, KYSE510 and KYSE520) [15] were obtained from DSMZ (Braunschweig, Germany), the German Resource Centre for Biological Material. Fifty pairs of primary ESCCs and their surrounding non-tumorous esophageal tissues were collected immediately after surgical resection at Linzhou Cancer Hospital (Henan, China). Samples used in this study were approved by the Committees for Ethical Review of Research involving Human Subjects at Zhengzhou University and Sun Yat-Sen University.

### Semiquantitative RT-PCR

Total RNA was extracted from cell lines and frozen ESCC tissues using the Trizol reagent (Invitrogen, Carlsbad, CA) according to the manufacture's instruction. Reverse transcripation of total RNA (2 μg) was done using SuperScript II reverse transcriptase (Invitrogen, Carlsbad, CA), and cDNA was subjected to PCR for a 30-cycle amplification with primers for GPR39Fw: 5'-GCCACCGGGGTCTCACTTGC-3' and GPR39Rv: 5'-GGCCGCAGCCATGATCCTCC-3'. *GAPDH *(Fw: 5'-CATGAGAAGTATGACAACAGCCT; Rv: 5'-AGTCCTTCCACGATACCAAAGT) was used as an internal control.

### Tissue Microarrays (TMA) and Immunohistochemistry (IHC)

A total of 300 formalin-fixed and paraffin-embedded ESCC tumor specimens were kindly provided by Linzhou Cancer Hospital (Henan, China). TMAs containing 300 pairs of primary ESCC tumor samples and their corresponding nontumourous tissues were constructed as described previously [16]. Standard streptavidin-biotin-peroxidase complex method was used for IHC staining [16]. Briefly, TMA section was deparaffinized, blocked with 10% normal rabbit serum for 10 min, and incubated with rabbit anti-human GPR39 polyclonal antibody (Abcam, 1:100 dilution) overnight at 4°C. The TMA section was then incubated with biotinylated goat anti-rabbit immunoglobulin at a concentration of 1:100 at 37°C for 30 min. All of the IHC staining results were reviewed independently by two pathologists. Positive expression of GPR39 was defined as the brown staining in the cytoplasm. The staining results for GPR39 were scored semiquantitatively. Intensity was estimated in comparison to the control and scored as follows: 0, negative staining; 1, weak staining; 2, moderate staining; and 3, strong staining. Scores representing the percentage of tumor cells stained positive were as follows: 0, <1% positive tumor cells; 1, 1-10%; 2, 10-50%; 3, 50-75%; and 4, >75%. A final score was calculated by adding the scores for percentage and intensity, resulting in scores of 0 and 2-7. A score of 0 was considered negative; 2-3 was considered weak; 4-5 was considered moderate; and 6-7 was considered strong. For statistical analysis, 0-3 were counted as low expression of GPR39, while 4-7 were counted as overexpression of GPR39.

### Tumorigenic function of GPR39

To test the tumorigenic function of GPR39, full-length GPR39 was PCR amplified, subcoloned into pcDNA3.1(+) vector (Invitrogen, Carlsbad, CA) and stably transfected into ESCC cell line KYSE30. Stable GPR39-expressing clones (GPR39-c1 and GPR39-c4) were selected for further study. Empty-vector transfected KYSE30 cells (Vec-30) were used as control.

For foci formation assay, 1 × 10^3 ^GPR39-expressing cells or Vec-30 cells were seeded into 6-well plate. After 7 days culture, surviving colonies (>50 cells/colony) were counted with 1% crystal violet staining. Triplicate independent experiments were performed. Colony formation in soft agar was performed by growing 1 × 10^4 ^cells in 0.4% Seaplague agar on a base of 0.6% agar in a 6-well plate. After 3 weeks, colonies consisted of more than 80 cells were counted and expressed as the means ± SD of triplicate within the same experiment. To perform cell growth assay, *GPR39*-expressing cells and control Vec-30 cells were seeded in 96-well plate at a density of 800 cells per well. The cell growth rate was measured using cell counting kit-8 kit (Dojindo, Japan) according to the manufacturer's instruction. Triplicate independent experiments were done.

### Flow cytometry assay

GPR39-c4 or Vec-30 cells were cultured in DMEM medium containing 10% FBS. Serum was withdraw from the culture medium when cells were 70% confluent. After 72 hrs, 10% FBS was added in the medium for an additional 8 hrs, Cells were fixed in 70% ethanol, stained with propidium iodide, and DNA content was analyzed by Cytomics FC (Beckman Coulter, Fullerton, CA).

### Tumor formation in nude mice

For *in vivo *experiment, stable GPR39-expressing KYSE30 cells or control Vec-30 cells (1 × 10^6^) in 200 μL serum-free DMEM (Life Technologies) were injected s.c. into the right and left flank of 4 week-old nude mice (5 mice for *GPR39*-c1 cells and 5 for *GPR39*-c4 cells), respectively. The tumor volume was calculated by the formula V = 0.5 × L × W^2 ^[17]. All experiments were done in accordance with institutional standard guidelines of Sun Yat-Sen University for animal experiments.

### Migration and invasion assays

For cell migration assay, GPR39-c4 cells or Vec-30 cells were grown to confluence and then mechanically scratched with a sterile pipette tip. Cells were rinsed with PBS and grown in culture medium for additional 24 hrs. The cell motility in terms of wound closure was measured by photographing at three random fields at time points 0 and 24 hr. For invasion assay, GPR39-c4 cells or Vec-30 cells were starved with serum free medium for 24 hrs before the assay. Cells (5 × 10^4^) were suspended in 0.5 ml serum-free medium and loaded on the upper compartment of invasion chamber coated with Matrigel (BD Biosciences). The lower compartment was filled with complete medium as chemoattractant. After 24 hrs, invasive cells were fixed, stained, and counted under a microscope. Triplicate independent experiments were done.

### F-actin staining

Cells grown on coverslips were washed three times in PBS, fixed in 4% paraformaldehyde for 20 min, and permeabilized with 0.1% Triton X-100 for 10 min. Cells were then stained with rhodamin-labeled phalloidin (Molecule Probes) in PBS containing 1% bovine serum albumin at room temperature for 30 min. After additional PBS washes, cells were counterstained with DAPI and photographed with a *Leica DMRA *fluorescence microscope (Rueil-Malmaison, France).

### RNA interference

Small interfering RNA (siRNA) (20 μM) against *GPR39 *(s6073; Ambion) was transfected into KYSE180 cells in 6-well plates using Lipofectamine 2000 Reagent (Invitrogen) according to the manufacturer's instructions. At 48 hrs after transfection, the effects of gene silencing were measured via RT-PCR.

### Western blot analysis

Western blot analysis was performed with the standard method with antibodies to GPR39, N-cadherin and GAPDH (Abcam, Cambridge Science Park, Cambridge, UK), cyclin D1, p21, CDK4 and CDK6 (Cell Signalling Technology, Frankfurt, Germany), and E-cadherin (Santa Cruz Biotechnology, Santa Cruz, CA).

### Statistical analysis

Statistical analysis was performed with the SPSS standard version 16.0 (SPSS Inc., Chicago, IL). The relationship between the expression of GPR39 protein and clinicopathologic characteristics was assessed by χ^2 ^test. Results expressed as mean ± SD were analyzed using the Student *t *test. Differences were considered significant when *P *< 0.05.

## Results

### GPR39 is frequently overexpressed in ESCC

**Figure 1:**
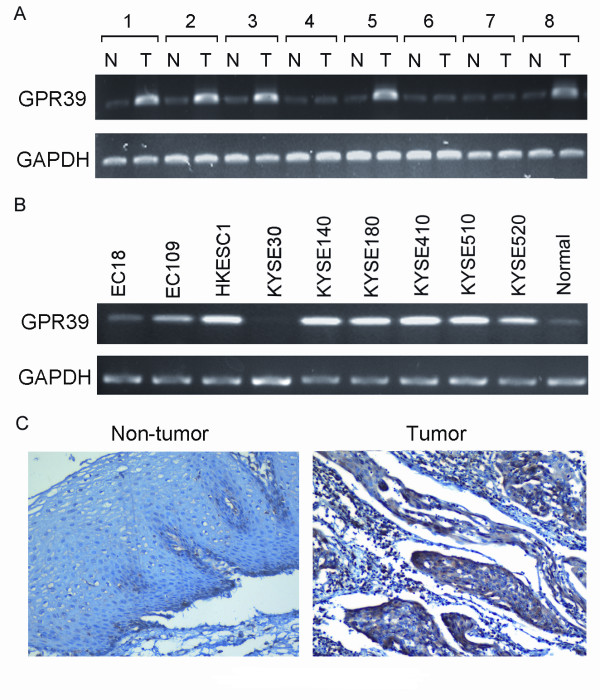
**Overexpression of GPR39 in ESCC**. *GPR39 *was frequently overexpressed in primary ESCCs (A) and ESCC cell lines (B) detected by RT-PCR. For primary ESCCs, expression of *GPR39 *in tumor tissues (T) was compared with their paired non-tumorous tissues (N). Normal esophageal tissue was used as a normal control. 18S rRNA was used as an internal control. (C) Representative of GPR39 expression in a pair of ESCC (right) and adjacent normal tissue (left) detected by immunostaining with anti-GPR39 antibody (brown). The slide was counterstained with hematoxylin (original magnification × 200).

Semi-quantitative RT-PCR was used to study the expression status of GPR39 in 50 primary ESCCs and 9 ESCC cell lines. Compared with their paired non-tumorous tissues, overexpression of GPR39 was detected in 27/50 (54%) of primary ESCCs (Figure 1A). Overexpression of GPR39 was also frequently detected in ESCC cell lines (HKESC1, KYSE140, KYSE180, KYSE410, KYSE510 and KYSE520; Figure 1B). GPR39 expression in protein level was further studied in 300 primary ESCCs by IHC using a tissue microarray. Informative IHC results were obtained from 207 pairs of ESCCs. Non-informative samples included lost samples, unrepresentative samples, samples with too few tumor cells, and samples with inappropriate staining; such were not used in data complication. The expression of GPR39 in normal epithelial cells was always negative or weak whereas strong positive staining of GPR39 was observed in 121/207 (58.5%) of informative ESCCs (Figure 1C).

### Clinical significance of GPR39 overexpression in ESCC

The correlation between the GPR39 overexpression and clinicopathologic features of ESCC including age (≤60 versus >60), gender (male versus female), tumor invasion (T stage: tumor depth; T3, T4 versus T1, T2), lymph nodes metastasis (N stage; N0 versus N1), TNM stage (I, IIa versus IIb, III-IV), was studied (Table 1). The results showed that overexpression of GPR39 was significantly associated with lymph node metastasis (*P *= 0.008) and advanced clinical stage (*P *= 0.004). No correlation was observed between GPR39 overexpression and age (*P *= 0.735), gender (*P *= 0.887), tumor differentiation (*P *= 0.846) and tumor invasion (*P *= 0.085).

**Table 1:** Association between GPR39 expression and clinical characteristics of ESCC patients (n = 207)

Clinicopathologic characteristics	GPR39 expression no. (%)		*P*
		
	Overexpression	Low expression	
Age (y)			
≤60	69 (59.5)	47 (40.5)	0.735
>60	52 (57.1)	39 (42.9)	
Sex			
Male	68 (57.6)	50 (42.4)	0.887
Female	53 (59.6)	36 (40.4)	
Tumor location			
Upper	22 (53.7)	19 (46.3)	0.762
Middle	82 (59.4)	56 (40.6)	
Lower	16 (61.5)	10 (38.5)	
Tumor cell differentiation			
Well	15 (53.6)	13 (46.4)	0.846
Moderate	76 (58.9)	53 (41.1)	
Poor	30 (60.0)	20 (40.0)	
Tumor invasion (T)			
T1	2 (25)	6 (75)	0.085
T2	44 (65.7)	23 (34.3)	
T3	75 (57.3)	56 (42.7)	
T4	1 (100)	0 (0)	
Lymph node metastasis (N)			
N0	59 (50.4)	58 (49.8)	0.008*
N1	62 (68.9)	28 (31.1)	
TNM stage			
I	1 (14.3)	6 (85.7)	0.004*
IIa	57 (52.3)	52 (47.7)	
IIb	15 (75.0)	5 (25.0)	
III-IV	48 (67.6)	23 (32.4)	

* Statistically significant (*P *< 0.05)

### Tumorigenic function of GPR39

**Figure 2:**
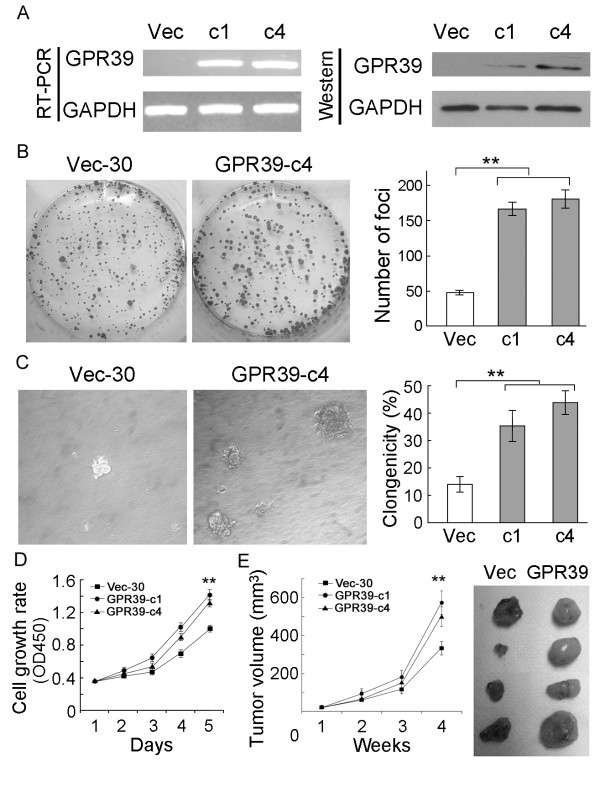
**Tumorigenic function of GPR39 in ESCC cells**. (A) Expression of GPR39 in *GPR39*-transfected KYSE30 cells was confirmed by RT-PCR (left) and Western blot analysis (right). c1 and c4 are two independent *GPR39*-expressing clones. Vec-30 represents empty vector-transfected KYSE30 cells. (B) Representative of foci formation in monolayer culture. Quantitative analyses of foci numbers were shown in the right panel. Values were the mean ± SD of at least three independent experiments. **P < 0.01; independent Student's *t*-test. (C) Representative of colony formation in soft agar. Percentage of colonies formed was summarized in the right panel. Values were the mean ± SD of at least three independent experiments. **P < 0.01. (D) Growth curves of *GPR39*-expressing cells were compared with Vec-30 cells by cell growth assay. The results were expressed as mean ± SD of at least three independent experiments. **P < 0.01. (E) Tumor growth curves of *GPR39*-expressing cells in nude mice were compared with Vec-30 cells by tumor xenograft experiment. The average tumor volume of *GPR39*-expressing cells vs Vec-30 cells was expressed as mean ± SD in 10 inoculated sites for each group of cells. **P < 0.01. (F) Representative examples of tumors formed in nude mice following injection of *GPR39*-expressing KYSE30 cells (right) and Vec-30 cells (left).

To investigate the tumorigenic potential of *GPR39*, *GPR39*-expression vector was stably transfected into KYSE30 cells with silenced *GPR39*. GPR39 mRNA and protein expression in these clones were confirmed by RT-PCR and Western blot analysis (Figure 2A). The tumorigenic function of *GPR39 *was assessed by both *in vitro *and *in vivo *assays including foci formation, colony formation in soft agar, cell growth rate assays and tumor xenograft experiment. Foci formation assay showed that the frequency of foci formation was significantly increased (*P *< 0.01) in *GPR39*-transfectants compared with control cells (Figure 2B). A similar result was shown in soft agar assay (*P *< 0.01, Figure 2C). Cell growth assay also revealed that the cell growth rates in *GPR39*-c1 and *GPR39*-c4 cells were significantly enhanced by *GPR39 *compared with Vec-30 cells (*P *< 0.01, Figure 2D). To further explore the *in vivo *tumorigenic ability of *GPR39*, tumor formation in nude mice was tested by injection of *GPR39*-c1 cells (n = 5) or *GPR39*-c4 cells (n = 5), whereas Vec-30 cells were used as controls. Tumor formation was observed in all tested animals. The results showed that the tumor growth curve of *GPR39*-overexpressing cells was significantly increased compared to Vec-30 cells (*P *< 0.01, Figure 2E).

### GPR39 promotes G_1_/S transition

**Figure 3:**
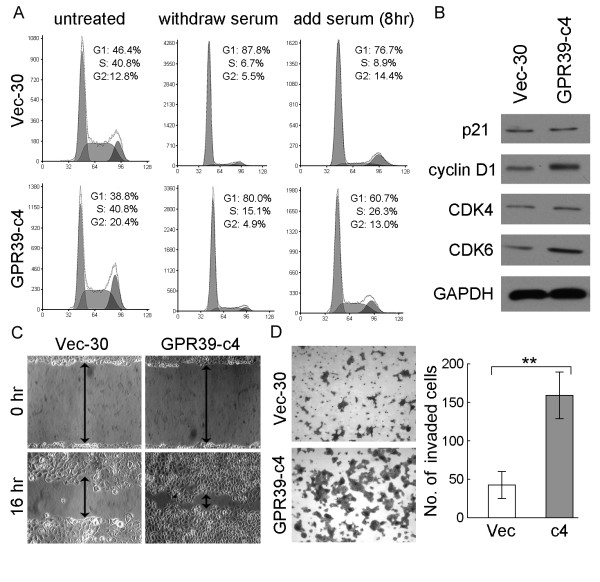
**GPR39 promotes G1/S transition and enhances cell motility**. (A) DNA content between *GPR39*-expressing cells and control Vec-30 cell were compared by Flow-cytometry. Untreated, cells were cultured in DMEM medium with 10% FBS; Withdraw serum, cells were cultured in DMEM medium without serum for 3 days; Add serum, cells were cultured again in DMEM medium with 10% FBS for 8 hr. (B) Expression of p21, cyclin D1, CDK4, and CDK6 were compared between *GPR39*-expressing cells (c4) and control Vec-30 cells by Western blot analyses. GAPDH was used as loading control. (C) The effect of *GPR39 *on cell migration was determined by wound-healing assay. During a period of 16 hr, the spreading speed of *GPR39*-expressing cells along the wound edge was faster than that in control Vec-30 cells. (D) Representative images showed the *GPR39*-expressing cells and Vec-30 cells that invaded through the matrigel. Number of invaded tumor cells was quantified in the right panel. *Columns*, mean of triplicate experiments; **P < 0.01.

To explore the mechanism underlying growth promotion by *GPR39*, the cell cycle distributions of *GPR39*-c4 and Vec-30 cells were determined by flow cytometry. Before treatment, the percentage of *GPR39*-c4 cells in G1 phase was obviously reduced in comparison with Vec-30 cells (38.37 ± 1.02% versus 45.87 ± 0.47%, *P *< 0.05; Figure 3A). After 3 days' serum starvation followed by addition of 10% serum for 8 hrs, the percentage of cells in S phase was significantly increased in *GPR39*-c4 cells compared to Vec-30 cells (26.43 ± 0.71% versus 8.97 ± 0.31%, *P *< 0.05; Figure 3A), suggesting that GPR39 was able to promote G1/S transition. To reveal the potential molecular mechanism of *GPR39 *in cell cycle promotion, expressions of several key cell cycle regulators including p21, cyclin D1, CDK4 and CDK6 were compared between *GPR39*-c4 and Vec-30 cells. Increased expression of cyclin D1 and CDK6, but not p21 and CDK4, were detected in *GPR39*-c4 (Figure 3B).

### GPR39 enhances cell motility and invasiveness of ESCCs

As the TMA result showed that overexpression of GPR39 was closely associated with ESCC metastasis, the effects of *GPR39 *on cell migration and invasion were studied by wound-healing and cell invasion assays. Wound-healing assay showed that that the ectopic expression of *GPR39 *could significantly increase cell migration ability in *GPR39*-transfected cells compared with empty-vector control (*P *< 0.05, Figure 3C). Matrigel invasion assay also found that the ectopic expression of *GPR39 *could significantly enhanced the invasiveness of ESCC cells, as demonstrated by a significant increase in the number of invaded cells (*P *< 0.01, Figure 3D), in *GPR39*-transfected cells compared with empty-vector control.

### GPR39 induces partial epithelial-mesenchymal transition (EMT)

**Figure 4:**
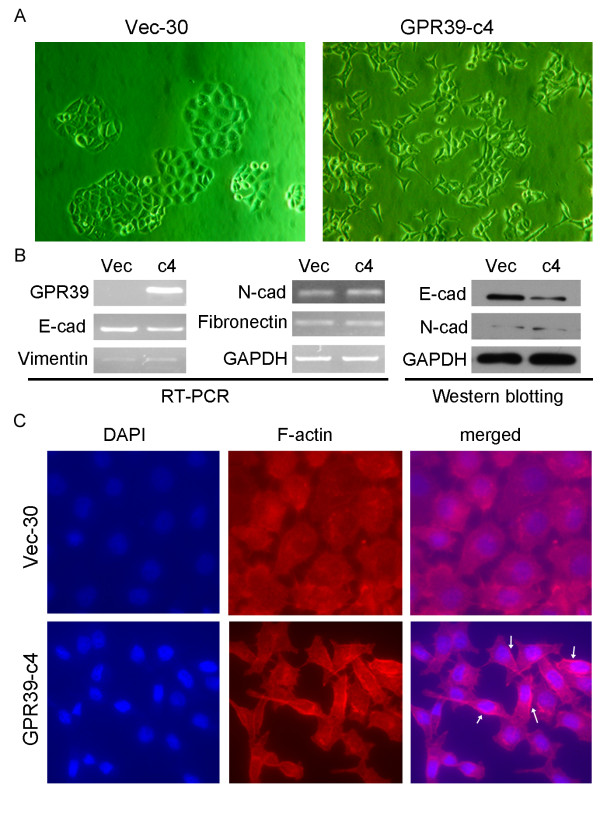
**GPR39 promotes cell mobility and invasion by inducing partial EMT and remodeling cytoskeleton**. (A) Representatives of cell morphology of GPR39-expressing cells and Vec-30 cells (original magnification × 200). (B) Expressions of epithelial markers E-cadherin and mesenchymal markers fibronectin, N-cadherin, and vimentin, were compared by RT-PCR or Western blotting analysis between GPR39-expressing cells and Vec-30 cells. GAPDH was used as loading control. (C) Representative images of F-actin staining. Formation of lamellipodia (indicated by arrows) was stimulated by GPR39 compared to control cells (magnification × 400).

In this study, we found that the cell morphology changed obviously after the transfection of *GPR39*. *GPR39*-transfected cells showed spindle shape and fibroblastic changes in monolayer culture, whereas empty vector-transfected cells, like KYSE30 parental cells, kept their cobblestone-like phenotype (Figure 4A). To determine whether the effect of *GPR39 *on cell motility was associated with EMT, expressions of several epithelial markers (E-cadherin, N-cadherin) and mesenchymal markers (vimentin, and fibronectin) were compared between GPR39-c4 and Vec-30 cells by RT-PCR and Western blot analysis. The results showed that E-cadherin was obviously down-regulated in *GPR39*-c4 cells; however, no obvious difference was observed in the expression of N-cadherin, vimentin and fibronectin between *GPR39*-c4 and Vec-30 cells (Figure 4B). These findings indicated that *GPR39 *increased cell motility was partially through the EMT.

### Overexpression of GPR39 induced lamellipodia formation

To further explore the molecular mechanism of *GPR39 *in regulating cancer invasion and metastasis, the role of *GPR39 *in the polymerized actin was investigated by phalloidin staining. The results showed that GPR39-expressing cells exhibited enhanced lamellipodia formation compared with control cells (Figure 4C), indicating that *GPR39 *could induce cytoskeleton remodeling to facilitate esophageal cancer cell migration and invasion.

### Silencing GPR39 expression by RNA interference (RNAi)

**Figure 5:**
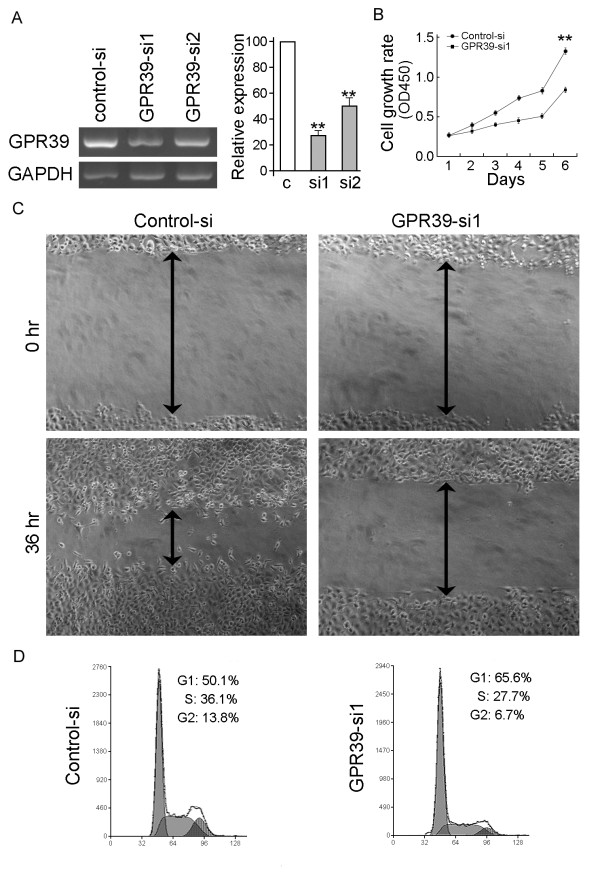
**Silencing of GPR39 expression suppresses tumorigenic ability of GPR39**. (A) GPR39 expression was efficiently decreased by the treatment of siGPR39 by RT-PCR. Relative expression level was measured by densitometer and summarized in the right panel. **P < 0.01. (B) Growth curve of KYSE180 cells treated with GPR39 siRNA was compared with control siRNA treated cells by cell growth assay. **P < 0.01. (C) Cell migration assay was used to compare the frequency of migratory cells between KYSE180 cells treated with control siRNA and GPR39 siRNA. (D) DNA content between control siRNA and GPR39 siRNA treated cells were compared by Flow-cytometry. *P < 0.05.

ESCC cell line KYSE180, which expresses a high level of endogenous *GPR39*, was used in the siRNA experiment. Two siRNAs targeting *GPR39 *(GPR39-si1 and GPR39-si2) were tested and the efficiency of *GPR39 *gene silencing was detected by RT-PCR. The result showed that the GPR39-si1 had a better silencing effect (Figure 5A). Silencing of *GPR39 *resulted in a significant inhibition of the cell growth rate (*P *< 0.01, Figure 5B) and migration (Figure 5C). DNA content analysis by flow cytometry showed that GPR39-si1 was able to inhibit the cell cycle at the G1/S checkpoint (Figure 5D). The percentage of cells in the S phase was significantly reduced in GPR39-si1-treated cells (27.23 ± 1.26%) compared with that in control-si-treated cells (35.13 ± 1.12%; *P *< 0.05). These findings further supported that the tumorigenic function of *GPR39 *was through its role in promoting cell proliferation and motility.

## Discussion

Many G protein-coupled receptors (GPCRs) have been found to play critical roles in the development and progression of cancer, including malignant transformation [18,19], tumor growth and survival [20,21], as well as invasion and metastasis [22,23]. Herein, we report that one of the G protein-coupled receptors, GPR39, is frequently overexpressed in human ESCC. To our knowledge, this is the first illustration that GPR39 contributes to the development and progression of ESCC. In the present study, the tumorigenic function of GPR39 was demonstrated by both in vitro and in vivo assays. Functional studies showed that GPR39 could effectively promote ESCC cancer cell growth, increase foci formation and colony formation and enhance tumor formation in nude mice. A recent study suggested that zinc could be a ligand capable of activating the GPR39 receptor [11]. Interestingly, zinc deficiency along with its associated increased cell proliferation can be tumorigenic in the rat esophagus [24,25]. Our study also provided evidence that ectopic expression of GPR39 increased ESCC cancer cell growth, indicating involvement of the GPR39 receptor in the tumorigenesis of esophageal cancer. However, whether GPR39 signaling is activated by zinc in esophageal carcinogenesis needs to be further investigated. Further study revealed that overexpression of GPR39 in esophageal cancer cells KYSE30 promoted G1/S phase transition. We showed for the first time that GPR39 controls cell cycle progression through the activation of CDK6 and its activating protein, cyclin D1. G1/S phase transition is a major checkpoint for cell cycle progression and cyclin D1-CDK6 complex is one of the critical positive regulators during this transition [26,27]. On the other hand, we found that silencing of GPR39 expression could inhibit tumorigenicity in KYSE180 cells through the cell cycle arrest at G1/S checkpoint.

Another interesting finding of this study is the promoting effect of GPR39 on tumor metastasis in ESCC. Our data showed that overexpression of GPR39 could promote cell motility and invasiveness of ESCC cells *in vitro*. This mirrored the findings of GPR39 overexpression in human ESCC samples and its association with advanced clinical stage and lymph node metastasis of ESCC. Conversely, when we knocked down the endogenous GPR39 by RNAi in ESCC cells, the mobility of ESCC cells was significantly reduced, suggesting that GPR39 is closely involved in ESCC invasion and metastasis. Moreover, the observation of overexpression of GPR39 resulting in cell morphological alteration promoted us to further investigate its effect on EMT. We found that GPR39 has some impact on the EMT as shown by decreasing the epithelial molecule E-cadherin, an event critical in tumour invasion and a 'master' regulator of EMT. E-cadherin provides a physical link among adjacent cells and is crucial for the establishment and maintenance of polarity and the structural integrity of epithelia. Indeed, due to the physical and functional link between E-cadherin based complexes and cytoskeletal components, a change in the E-cadherin mediated adhesiveness leads to rearrangement of the cytoskeleton [28]. In view of this, we further explored the role of GPR39 in reorganization of the actin cytoskeleton. As expected, our result showed that GPR39 led to significant alterations on cytoskeleton by inducing the lamellipodia formation in *GPR39*-transfected ESCC cells. This finding was consistent to previous studies that some G protein-coupled receptors (GPCRs) were able to promote actin reorganization and result in cell shape changes and enhanced cell migration [13,29], indicating that GPR39 might directly alter the cytoskeleton to favor the tumor cell invasion and metastasis in ESCC.

In this study, we have also provided evidence that targeting of *GPR39 *with specific RNAi will reduce the oncogenic characteristics of ESCC tumor cells. To date, some G protein-coupled receptors (GPCRs) provide important practical options for preclinical research, clinical trials, and cancer treatment [30]. Therefore, consideration should be given to the development of novel therapeutics targeting GPR39 for use in GPR39-expressing ESCC tumors.

## Conclusions

In summary, our findings demonstrate that GPR39 plays an important role in ESCC development and progression via promoting cell proliferation, enhancing cell motility and invasiveness, regulating cytoskeleton and inducing EMT. A better understanding of the molecular mechanism of GPR39 in ESCC development and progression would provide novel therapeutic strategies to ESCC cancer patients.

## Abbreviations

EMT: epithelial mesenchymal transition; ESCC: esophageal squamous cell carcinoma; GPCR: G protein-coupled receptor; siRNA: small interfering RNA; TMA: tissue microarray; TSG: tumor suppressor gene; L: length; V: volume; W: width.

## Competing interests

The authors declare that they have no competing interests.

## Authors' contributions

FX and HL performed the experimental procedures with support from YZ, YQ, YD, TZ, LC, CN, TH and YL. FX, LF and XYG were responsible for experimental design, interpretation of the results and writing the manuscript. All authors read and approved the final manuscript.

## Pre-publication history

The pre-publication history for this paper can be accessed here:

http://www.biomedcentral.com/1471-2407/11/86/prepub
